# Prevalence of Osteoporosis and Its Effect on Residual Ridge Resorption in Postmenopausal Females of Rural Central Indian Region

**DOI:** 10.7759/cureus.61699

**Published:** 2024-06-04

**Authors:** Sheetal R Khubchandani, Sweta G Pisulkar, Surekha A Dubey

**Affiliations:** 1 Department of Prosthodontics and Crown and Bridge, Sharad Pawar Dental College and Hospital, Wardha, IND

**Keywords:** women, residual ridge resorption (rrr), bone mineral density (bmd), body mass index (bmi), osteoporosis

## Abstract

Aim: To determine the correlation between body mass index (BMI), bone mineral density (BMD), and residual ridge resorption (RRR) in postmenopausal females and the effect of osteoporosis on RRR.

Materials and methods: A study was conducted with 60 postmenopausal female individuals. BMI was calculated using the weight and height of the patient using a formula. BMD was assessed and graded using a T-score. RRR was determined using the Tallgren method.

Results: Most individuals showed a higher BMI (63.33%), which is in the overweight or obese category. BMD was lower in approximately 68.33% of patients, and RRR was significantly higher in about 60% of total patients.

Conclusion: The higher the BMI values, the lesser the BMD and the higher the RRR.

## Introduction

Osteoporosis is a systemic bone disease that is often associated with vitamin D deficiency. It is characterized by decreased bone mass, and the microstructure of the bone is also affected. Bone density decreases, affecting the overall bone quality. This leads to fragile bones, which become prone to fractures [1]. Vitamin D deficiency is the most common nutritional deficiency, but unfortunately, it is often underdiagnosed and, therefore, not promptly treated. It is widespread in developing countries such as India, irrespective of the demography [2,3].

Osteoporosis affects all the bones, including the alveolar bone. The bone that remains after teeth extraction or natural exfoliation of teeth is called the residual ridge. The resorption of such bone is termed residual ridge resorption (RRR). RRR is the most important parameter considered for patients receiving any removable, fixed, complete, or partial prosthesis [4,5]. It is a chronic, progressive, complex, irreversible, and crippling illness. There is osteoclastic activity in the microscopic pathology of residual ridge repossession, particularly on the crest's outside surface. The edentulous mandibular region experiences bone loss, which leads to the most dramatic alterations. However, it varies from patient to patient and from one location to another within the same patient.

Local factors related to RRR include tooth extraction, quality, quantity, ridge shape, muscle attachment shape, edentulousness, and bite stress from the denture. The systemic factors comprise the patient’s age, gender, calcium and phosphorous metabolism disorders, and hormonal imbalance [6,7]. The research has hypothesized that systemic variables, such as body mass index (BMI), generalized bone mineral density (BMD), and osteoporosis, also significantly affect RRR. Women experience age- and hormone-related bone loss more frequently than men do, especially after menopause due to ovarian shrinkage and a decline in estrogen levels. Its exact origin is unknown, but many local and systemic factors are related to RRR [8]. The literature also describes BMI as one of the predisposing variables for atrophic jaw bones, highlighting the fact that patients with decreased BMD do not necessarily have atrophic jaw bones or the reverse. Therefore, patients with smaller anatomical bones and a finer body structure may exhibit more jaw bone resorption symptoms than patients with higher body mass indices. The study aimed to find a correlation between BMI, BMD, and RRR.

## Materials and methods

It was an observational study carried out at the Department of Prosthodontics and Crown and Bridge, Sharad Pawar Dental College and Hospital, Sawangi (Meghe), Wardha, a part of Datta Meghe Institute of Higher Education and Research (deemed to be a university) in collaboration with Acharya Vinoba Bhave Rural Hospital, Sawangi (Meghe), Wardha. The study design is an observational study with a duration of six months (short-term study). The total number of subjects included in the sample is 60 female patients. The inclusion criteria are as follows: postmenopausal and completely edentulous female patients from the central Indian region, aged 50 to 85 years, reported to the Department of Prosthodontics to have conventional complete dentures for both upper and lower jaws. All these patients should have at least a 2-year period after extraction of the last tooth.

Steps

1. Determination of BMD: Using quantitative computed tomography (QCT), the BMD in the lumbar region L2-L4 and the femoral neck was ascertained. Following the collection of QCT results, patients were assigned to one of three groups: normal BMD, osteopenia, or osteoporosis, based on the worst finding from both. The World Health Organization T-score scale (for dual-energy X-ray absorptiometry) can also be applied to QCT. This scale indicates the number of standard deviations above or below the mean for a healthy adult patient of the same sex, ethnicity, and age (30 years). It was used to assign patients to their respective groups. Normal BMD has a score of greater than or equal to -1.0, osteopenia has a score of -1.0 to -2.5, and osteoporosis has a score of ≤-2.5.

2. Determination of BMI: The BMI (kg/m^2^) was calculated using the patient's weight (in kg) and height (in m).

3. Determination of RRR: The RRR of edentulous jaws was ascertained through radiographic and clinical investigations. The Tallgren method radiological measurement (Y1) was utilized to assess the symphysis of the jaw, measuring from the menton to the crest of the residual ridge in the digital lateral cephalogram of each patient using the Pantomograph Trophycan C. Based on diagnostic casts of anatomic impressions, the degree of edentulous RRR was classified as low, moderate, or severe clinically.

Statistical analysis: Descriptive and analytical statistics were done. The correlation coefficient was calculated using Pearson’s correlation test. The level of significance was kept at p < 0.05. The software used was Statistical Package for Social Sciences version 24.0 (IBM Corporation, Chicago, IL, USA).

## Results

The distribution of patients based on the average T-score shows that 68.33% (41) were osteoporotic, 19.33% (11) were osteopenic, and only 13.33% (8) were normal (Table 1). BMI measurements revealed only 8.33% (5) had a normal weight, while 28.33% (17) were overweight and 63.33% (38) were in the obese category (Table 2). RRR readings showed that 60% (36) had severe resorption, 23.33% (14) had moderate resorption, and 16.67% (10) had minimal resorption (Table 3). The above-mentioned results justify the fact that osteoporosis is prevalent in elderly edentulous patients who are overweight or obese, and they show moderate to severe resorption.

**Table 1:** Distribution of patients according to BMD BMD: bone mineral density

BMD	No. of patients	Percentage
Normal	8	13.33%
Osteopenic	11	19.33%
Osteoporotic	41	68.33%
Total	60	100%

**Table 2:** Distribution of patients according to BMI BMI: body mass index

BMI	No. of patients	Percentage
Normal	5	8.33%
Overweight	17	28.33%
Obese	38	63.33%
Total	60	100%

**Table 3:** Distribution of patients according to RRR RRR: residual ridge resorption

RRR	No. of patients	Percentage
Minimal (0)	10	16.67%
Moderate (1)	14	23.33%
Severe (2)	36	60%
Total	60	100%

A statistically significant (p < 0.001) strong negative correlation R = -0.793 was found between the BMI and BMD values. This means that the higher the BMI, the lesser the BMD values. A statistically significant (p < 0.001) strong positive correlation R = 0.792 was found between the BMI and RRR values. This means that the higher the BMI, the lesser the BMD and the higher the RRR (Table 4).

**Table 4:** Correlation of BMD and RRR values BMD: bone mineral density; RRR: residual ridge resorption #P value derived from Person’s correlation test †Significant at p < 0.05

Groups	N	R value	P value^#^
BMD	60	-0.793	<0.001^†^
RRR	36	0.792	<0.001^†^

## Discussion

Osteoporosis is correlated with several characteristics. We looked into several factors, including RRR in postmenopausal females, BMD to measure osteoporosis, and BMI. Montazerifar et al. [8] investigated how postmenopausal women's BMD was impacted by their age, weight, and BMI. The results showed that reduced bone mass was more common in older women with low BMI. Although age, body weight, and BMI are significant indicators of BMD, they are not the only variables influencing bone loss. As a result, it is advised to evaluate additional risk variables on a bigger patient base. One of those risk variables was found to be obesity in the current investigation. A woman may have low BMD even if her BMI is high.

Additionally, Majumder and Harun [9] assessed the alterations in alveolar bone among postmenopausal women with osteopenia and osteoporosis. They found a considerable correlation between the BMD of systemic skeletal bone and alterations in the alveolar bone of postmenopausal women. This coupled relationship can serve as a simple diagnostic tool for advancements in osteoporotic conditions. Finkelstein et al. [10] examined bone loss rates at each transition stage and the major factors that modify those rates. He came to the conclusion that bone loss begins to accelerate during late perimenopause and persists at a comparable rate during the early postmenopausal years. While ethnicity itself does not significantly impact the rate of menopausal BMD loss, body weight does. Healthcare professionals should consider these details when determining when to screen women for osteoporosis.

Rajan et al. [11] concluded that osteoporosis is the major cause of fractures, contributing significantly to morbidity and mortality. Therefore, studies should be conducted at the cellular level to diagnose and determine the etiology for proper therapy. Therapy can be provided through nonpharmacological and pharmacological means, as well as through lifestyle modifications. Khinda et al. [12] concluded that about 30%-45% of women after menopause are found to be osteopenic or osteoporotic. They have also studied individual risk factors, and independent risk factors are exposed. They also concluded that higher BMI can reduce the risk of osteoporosis. They also suggested that every woman should undergo thorough screening and preventive measures should be taken before the condition worsens. The findings are coincident with the study by Imran et al., who also concluded that the prevalence of osteoporotic fractures was more than 82% [13].

Both men and women are frequently affected by osteoporosis and the resulting fragility fractures, which pose a serious and expanding hazard to world health. Men have a higher risk of incapacitation or death from osteoporotic fractures because women experience primary osteoporosis more frequently [14]. Schini et al. advocated a fracture risk assessment tool for assessing fracture risk. It is cost-effective and also determines BMD [15]. Ramchand and Leder assessed the relative benefits of antiresorptive, dual-acting, and bone-forming pharmaceuticals when used in particular orders. They also suggested that antiresorptive treatments should be taken first, followed by dual-acting or bone-forming therapies [16]. Alisherovna et al. suggested that the severity of the menopausal syndrome depends on the severity of the menopause. It also includes manifestations such as coronary heart disease, which is again an effect of higher BMI or obesity [17].

The study's limitations are that it is a short-term project. A long-term study can be done with a larger sample size to get more predictable results. The investigation used here was an orthopantomogram, which is a two-dimensional image. 3D imaging, like computed tomography scans or cone-beam computed tomography scans, can be used in further studies.

## Conclusions

There is little, if any, change in BMD in women in midlife before or early perimenopause. Early in the postmenopausal years, BMD loss continues to be rapid and accelerates significantly in the late perimenopause. One significant factor influencing the rates of BMD loss during the menopause transition is body weight. According to these results, healthcare professionals ought to think about doing osteoporosis screenings on women who are approaching the end of the menopause, especially if they are relatively light-weighted.
